# Rumen Epithelial Development- and Metabolism-Related Genes Regulate Their Micromorphology and VFAs Mediating Plateau Adaptability at Different Ages in Tibetan Sheep

**DOI:** 10.3390/ijms232416078

**Published:** 2022-12-16

**Authors:** Yuzhu Sha, Yanyu He, Xiu Liu, Shengguo Zhao, Jiang Hu, Jiqing Wang, Shaobin Li, Wenhao Li, Bingang Shi, Zhiyun Hao

**Affiliations:** 1 College of Animal Science and Technology/Gansu Key Laboratory of Herbivorous Animal Biotechnology, Gansu Agricultural University, Lanzhou 730070, China; 2 School of Fundamental Sciences, Massey University, Palmerston North 4410, New Zealand; 3 Academy of Animal Science and Veterinary Medicine, Qinghai University, Xining 810000, China

**Keywords:** Tibetan sheep, rumen epithelial, VFAs, micromorphology, immune

## Abstract

The rumen is an important hallmark organ of ruminants and plays an important role in the metabolism and immune barrier of Tibetan sheep on the Plateau. However, there are few studies on rumen development and metabolism regulation in Tibetan sheep at different ages. Here, we comprehensively analyzed the immune function, fermentation function, rumen epithelial micromorphology and transcriptome profile of Tibetan sheep at different ages. The results showed that the concentration of IgG decreased and the concentration of IgM increased with age (*p* < 0.05), and the highest concentration of IgA was observed at 1.5 and 3.5 years of age. In terms of rumen fermentation characteristics, VFAs of 4-month-old lambs were the highest, followed by VFAs and NH_3_-N of Tibetan sheep at 3.5 years of age. Hematoxylin-eosin staining and transmission electron microscopy section examination of rumen epithelial tissue showed that the rumen papilla width increased with age (*p* < 0.001), the thickness of the stratum corneum decreased, the cells in the stratum corneum showed accelerated migration and the thickness of the rumen muscle layer increased (*p* < 0.001). Desmosomal junctions between the layers of rumen epithelium increased at 1.5 and 3.5 years old, forming a compact barrier structure, and the basal layer had more mitochondria involved in the regulation of energy metabolism. RNA-seq analysis revealed that a total of 1006 differentially expressed genes (DEGs) were identified at four ages. The DEGs of Tibetan sheep aged 4 months and 6 years were mainly enriched in the oxidation–reduction process and ISG15-protein conjugation pathway. The 1.5 and 3.5-year-olds were mainly enriched in skeletal muscle thin filament assembly, mesenchyme migration and the tight junction pathway. WGCNA showed that DEGs related to rumen microbiota metabolite VFAs and epithelial morphology were enriched in “Metabolism of xenobiotics by cytochrome P450, PPAR signaling pathway, Butanoate metabolism pathways” and participated in the regulation of rumen epithelial immune and fermentation metabolism functions of Tibetan sheep at different ages. This study systematically revealed the regulatory mechanism of rumen epithelial development and metabolism in the plateau adaptation of Tibetan sheep, providing a new approach for the study of plateau adaptation.

## 1. Introduction

Tibetan sheep (*Ovis aries*) live in the Qinghai–Tibet Plateau region at 2500 m~5000 m above sea level and are primitive sheep breeds that live under harsh habitat conditions (extreme cold, low oxygen, strong ultraviolet light and nutrition stress in the cold season). They are one of the main economic resources for local herdsmen and provide meat, wool, milk and other products [1], and their special adaptability to the plateau environment is an important factor in determining their production performance and production level. Under the traditional feeding and management mode in the pastoral areas of the Qinghai–Tibet Plateau, Tibetan sheep graze on plateau pastures year-round, feeding on natural forage as the main source of nutrients. They generate energy for survival and reproduction from crude forage fiber through a strong rumen fermentation function, adapting to the extremely harsh plateau environment [2] with a certain genetic adaptation mechanism. Genetic adaptation is the core of evolutionary biology. In high-altitude areas, the adaptive evolution of ruminants will affect the expression of genes related to energy metabolism, which is closely related to rumen metabolism [3]. Our previous studies also found that Plateau Tibetan sheep regulate nutrient absorption and immune barrier function through rumen fermentation interacting with epithelial genes [4]. The rumen is the hallmark organ of ruminants and plays an important role in host metabolism, immunity and health [5]. The rumen epithelium consists of leaf nipples; it is the absorption structure in the rumen and prevents microbiota and/or toxin invasion of the epithelial barrier [6], which is composed of microbiota barriers, physical barriers and the immune barrier [7]. These findings show that the structure and related gene expression in the rumen epithelium play an important role in energy metabolism and the immune barrier of ruminants.

Rumen microbiota ferment fiber and other substances in forage into volatile fatty acids (VFAs), approximately 50%~85% of which are directly transported and absorbed by rumen epithelial cells as the main energy source for ruminants [8]. The absorption process of rumen epithelium is not only dependent on its morphological structure (papillary surface area, etc.), but is also related to the expression characteristics of related transporter genes/proteins [9,10]. VFAs can regulate a variety of physiological functions in the rumen [11], participate in the development and morphological changes of the rumen epithelium [12], and promote the proliferation and renewal of epithelial cells [13]. Studies on the rumen epithelial transcriptome [12,14] have found that the morphological structure of the rumen epithelium is related to gene function; for example, cell development, epithelial cell proliferation [15], papilla size and surface area [16] and tight junctions [17] are all affected by target gene function, and the transcriptome characteristics of the sheep gastrointestinal tract are also related to immunity and epithelial metabolism [18]. Furthermore, VFAs are also involved in the body’s metabolism, immune barrier and other processes. In addition to being an important energy substance for ruminants [19,20], VFAs also participate in the regulation of rumen epithelial growth, leptin levels, insulin secretion and the immune response [21,22]; for example, VFAs regulate the immune response through *GPCR41* [23]. Studies have found that 36 genes related to VFA transport and absorption are significantly upregulated in the rumen epithelium of ruminants at high altitude, revealing that the adaptability of animals at high altitude is associated with rumen phenotypic metabolism [3]. The rumen has a unique gene expression profile, and genes in the rumen epithelium are widely distributed in different tissues, demonstrating the complexity of rumen structure and the importance of regulatory changes in evolution. Other studies have found that genes with relatively high expression in the rumen show functional enrichment in ketone body metabolism, microbial community regulation and epithelial absorption and they participate in various biological processes [24]. All these studies indicate that the morphological structure and gene expression of the rumen epithelium are involved in the development and metabolic regulation of the body, so that animals can adapt to the corresponding environment. Therefore, it is of great significance to study the adaptation mechanism related to rumen epithelial development and metabolism in Tibetan sheep to improve their altitude adaptability. However, Tibetan sheep of different ages have different adaptability to plateau environments. There are few reports on the development and metabolism of the rumen epithelium in Tibetan sheep at different ages, and the regulation of rumen epithelial development and metabolism in plateau adaptation is also unclear. Therefore, the serum immune level, rumen fermentation function (VFAs, NH_3_-N), rumen epithelial micromorphology (HE/TEM) and transcriptomics of Tibetan sheep at different ages were analyzed in this study. This report aims to clarify the differences in rumen development and metabolism of Tibetan sheep at different ages, provide a reference for the feeding and management of Tibetan sheep at different ages and reveal the adaptive characteristics of rumen epithelial development and metabolic pathways in response to the plateau environment by synthetic analysis to provide new ideas for the study of plateau adaptation.

## 2. Results

### 2.1. Serum Immune Levels

**Figure 1:**
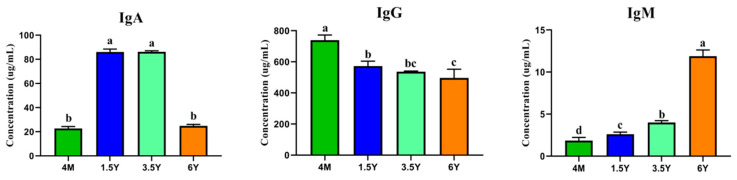
Serum immune indices at different ages. Note: Different lowercase letters above the column indicate significant differences, *p* < 0.05. 4M: 4 months; 1.5Y: 1.5 years; 3.5Y: 3.5 years; 6Y: 6 years.

As shown in Figure 1, serum immune levels of Tibetan sheep at different ages were different, and IgA concentration was the highest in 1.5 years and 3.5 years, which was different from 4 months and 6 years (*p* < 0.05). The concentration of IgG decreased with age, and the concentration of 4 months was higher than that of other ages (*p* < 0.05); IgM concentration increased with age (*p* < 0.05).

### 2.2. Rumen Fermentation Characteristics

As shown in Table 1, the rumen VFAs content of Tibetan sheep at different ages varied, and the total VFAs content of 4 months Tibetan sheep was higher than that of other ages (*p* < 0.001), followed by 3.5 years, which was higher than 1.5 years and 6 years. The contents of acetic acid, propionic acid, isobutyric acid, butyric acid and isovaleric acid were higher in the 4 months group than in other age groups (*p* < 0.001). Similarly, the contents of acetic acid, propionic acid, isobutyric acid, butyric acid and isovaleric acid were higher in 3.5 years than in 1.5 years and 6 years (*p* < 0.05), while the contents of VFAs were decreased in 6 years (*p* < 0.05). The A/P value at 1.5 years was higher than that at 4 months and 3.5 years (*p* = 0.023), the NH_3_-N content at 3.5 years was the highest and higher than that at other ages (*p* < 0.001).

### 2.3. Rumen Epithelial Development Structure

#### 2.3.1. Histological Characteristics of the Rumen Epithelium (HE)

**Figure 2:**
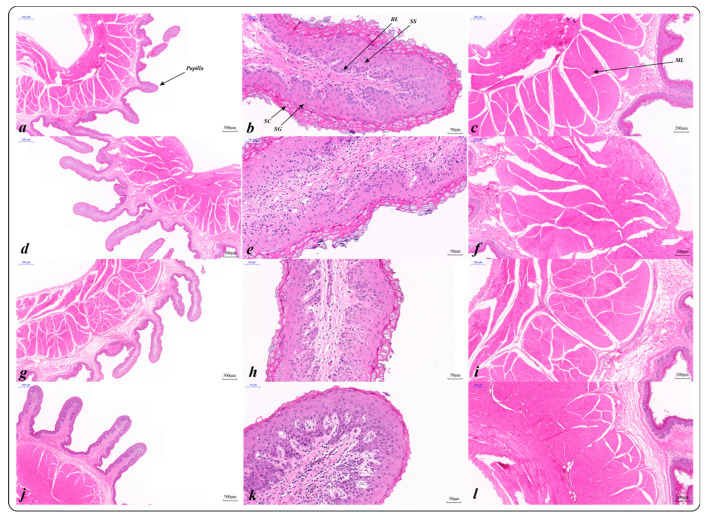
Histological characteristics of rumen epithelium of Tibetan sheep at different ages: 4 months (**a**–**c**); 1.5 years (**d**–**f**); 3.5 years (**g**–**i**); 6 years (**j**–**l**); SC: stratum corneum; SG: stratum granulosum; SS: stratum spinosum; BL: basal layer; ML: muscular layer.

The rumen epithelial histological structure of Tibetan sheep of different ages was different (Table 2, Figure 2). The width of rumen papilla increased with age (*p* < 0.001), except for 4 months papilla length, and there was no significant difference in other age groups (*p* < 0.001). As shown in Figure 2, the rumen epithelial layer is divided into the stratum corneum (SC), stratum granulosum (SG), stratum spinosum (SS) and basal layer (BL). With increasing of age, the thickness of the stratum corneum decreases, and the stratum corneum partially falls off at 6 years, making the thickness lower than that at other ages (*p* < 0.001). The thickness of the stratum granulosum at 1.5 years was lower than that at other ages (*p* < 0.001), there was no difference in stratum spinosum thickness among different age groups (*p* = 0.265), and the basal layer thickness at 6 years was higher than that at other ages (*p* = 0.002). In addition, rumen muscularis thickness also increased with age (*p* < 0.001).

#### 2.3.2. Ultrastructural Characteristics of Rumen Epithelium (TEM)

**Figure 3:**
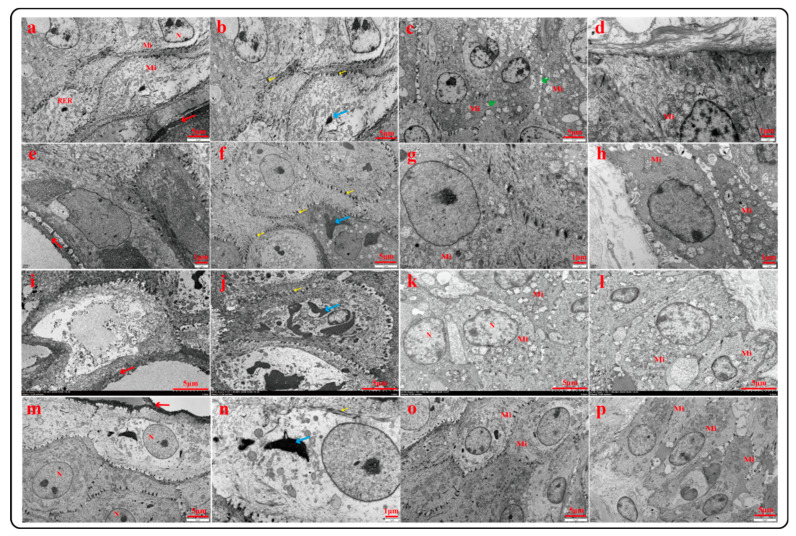
Ultrastructure of rumen epithelium of Tibetan sheep at different ages: 4 months (**a**–**d**); 1.5 years (**e**–**h**); 3.5 years (**i**–**l**); 6 years (**m**–**p**); nucleus (N), mitochondria (Mi), stratum corneum (↓), keratin granules (↓), desmosomal junctions (↓), intercellular space (↓).

To further observe the ultrastructure of the rumen epithelium, the rumen epithelium of Tibetan sheep of different ages was analyzed by transmission electron microscopy (TEM). As shown in Figure 3, the ultrastructure of each layer of the rumen epithelium varies with age. With increasing age, the stratum corneum cells showed an accelerated migration state, and the intercellular space gradually increased. It was obvious that the stratum corneum fell off at 6 years, which was consistent with the previous light microscope observation results, and the stratum corneum cells in 4 months were more compact. With increasing age, the connection between the stratum corneum and stratum granulosum becomes loose, a large number of keratinized granules exist and the number of granules increases. There were a large number of desmosomal junctions between cells in the granulosa layer, and the desmosomal junctions were the most abundant at 1.5 years and 3.5 years, thus forming a compact structure. It was found that there were more mitochondria in the stratum spinosum and basal layer, with the highest number at 1.5 and 3.5 years of age and less at 6 years of age. In addition, the basal layer cells tended to migrate with increasing age.

### 2.4. mRNA Expression Profile of the Rumen Epithelium

#### 2.4.1. Differential mRNA Analysis

**Figure 4:**
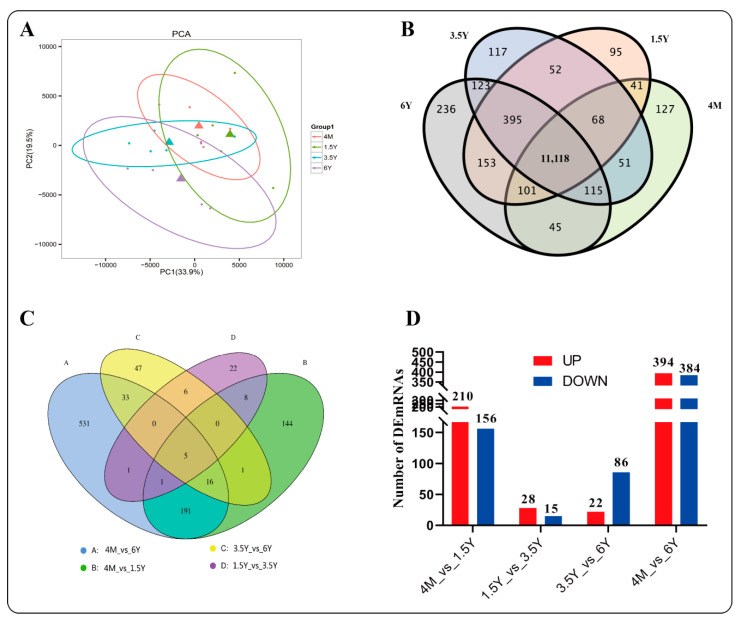
Differential mRNA analysis. (**A**): PCoA diagram; (**B**): Venn map of expressed genes; (**C**): differential gene Venn map; (**D**): up-down differential gene analysis. 4M: 4 months; 1.5Y: 1.5 years; 3.5Y: 3.5 years; 6Y: 6 years.

A total of 134.34 Gb of clean data were obtained by Seq-RNA analysis of rumen epithelial tissues of Tibetan sheep of different ages. The amount of clean data for each sample was 5.82 Gb, and the percentage of Q30 bases was more than 92.21%. The efficiency of sequence alignment with the reference genome was 94.04%~95.08%. PCA analysis showed that there were some differences in gene expression among different ages (Figure 4A). A total of 11,666/12,023/12,039/12,286 genes were identified at the four ages, among which 11,118 genes were coexpressed (Figure 4B). Fold change ≥ 2 and FDR < 0.01 were used as the screening criteria, and a total of 1,006 differentially expressed genes (DEGs) were screened, for which the 4 months_vs._1.5 years, 1.5 years_vs._3.5 years, 3.5 years_vs._6 years and 4 months_vs._6 years groups were compared and 366, 43, 108 and 778 DEGs were identified, respectively (Figure 4C,D). In addition, 144, 22, 47 and 531 unique DEGs were identified in the four age groups, as well as five differentially coexpressed genes (*gene-ACTA1, gene-ADAMTS4, gene-NOS2, gene-PPM1J, gene-SULT1C2*).

#### 2.4.2. GO and KEGG Functional Enrichment Analysis of DEGs

**Figure 5:**
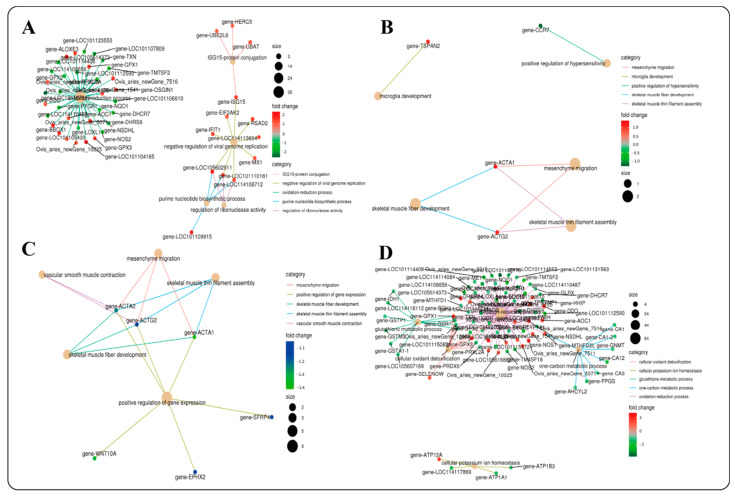
BP functional enrichment diagram. (**A**): 4 months_vs._1.5 years; (**B**): 1.5 years_vs._3.5 years; (**C**): 3.5 years_vs._6 years; (**D**): 4 months_vs._6 years. Note: The red dots represent upregulated genes, and the green dots represent downregulated genes.

In the four comparison groups, 328 (4 months_vs._1.5 years), 38 (1.5 years_vs._3.5 years), 102 (3.5 years_vs._6 years) and 694 (4 months_vs._6 years) DEGs were annotated in the GO database. Most of them were enriched in biological processes (BP) and cellular components (CC) (Figure S1). These genes were mainly enriched in cellular process, binding, metabolic process, biological regulation, membrane part and response to stimulus, etc., and the differentially enriched genes were different among all groups. Further enrichment analysis of biological processes (BP) showed that differentially expressed genes were mainly enriched in negative regulation of viral genome replication, oxidation-reduction process, purine nucleotide biosynthetic process, ISG15-protein conjugation and regulation of ribonuclease activity in the 4 months_vs._1.5 years group (Figure 5A). In the 1.5 years_vs._3.5 years group, skeletal muscle thin filament assembly, mesenchyme migration, skeletal muscle fiber development, positive regulation of hypersensitivity and microglia development were enriched (Figure 5B). In the 3.5 years_vs._6 years group, skeletal muscle thin filament assembly, mesenchyme migration, vascular smooth muscle contraction, positive regulation of gene expression and skeletal muscle fiber development were enriched (Figure 5C). In the 4 months_vs._6 years group, oxidation–reduction process, glutathione metabolic process, one-carbon metabolic process, cellular oxidant detoxification and cellular potassium ion homeostasis were enriched (Figure 5D).

**Figure 6:**
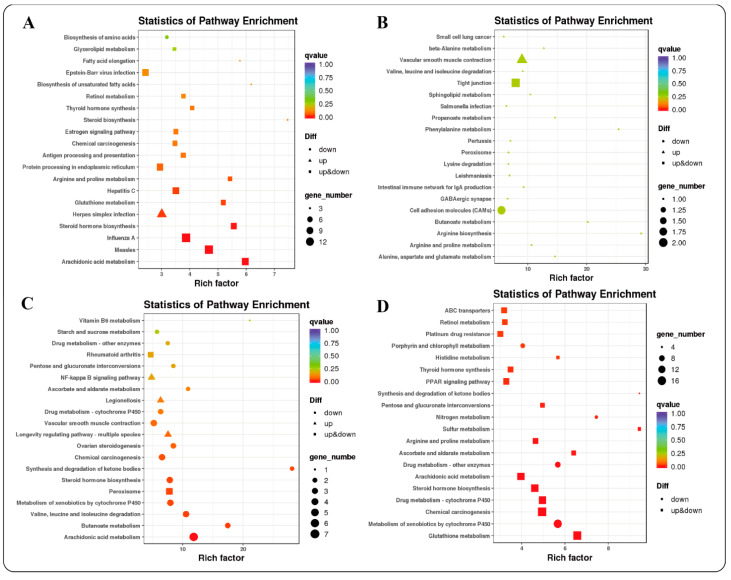
Functional enrichment analysis of KEGG. (**A**): 4 months_vs._1.5 years; (**B**): 1.5 years_vs._3.5 years; (**C**): 3.5 years_vs._6 years; (**D**): 4 months_vs._6 years.

KEGG functional enrichment analysis found that 238 (4 months_vs._1.5 years), 28 (1.5 years_vs._3.5 years), 84 (3.5 years_vs._6 years) and 537 (4 months_vs._6 years) differentially expressed genes were enriched in the four groups. The 4 months_vs._1.5 years group was mainly enriched in arachidonic acid metabolism (ko00590), glutathione metabolism (ko00480), arginine and proline metabolism (ko00330) and protein processing in the endoplasmic reticulum pathway (ko04141) (Figure 6A), and this has the function of protecting animals from lipid peroxide damage. The 1.5 years_vs._3.5 years group was mainly enriched in vascular smooth muscle contraction (ko04270), tight junctions (ko04530) and cell adhesion molecules (CAMs) (ko04514), and this has the function of increased peristalsis and contraction force (Figure 6B). The 3.5 years_vs._6 years group was mainly enriched in arachidonic acid metabolism, butanoate metabolism, metabolism of xenobiotics by cytochrome P450, steroid hormone biosynthesis and longevity-regulating pathway (multiple species) (Figure 6C), and this has the function of reducing metabolic level and changing immune function. The 4 months_vs._6 years group was mainly enriched in glutathione metabolism (ko00480), metabolism of xenobiotics by cytochrome P450 (ko00980), arachidonic acid metabolism (ko00590), arginine and proline metabolism (ko00330), PPAR signaling pathway (ko03320) and reduction of immune function (Figure 6D).

### 2.5. WGCNA

**Figure 7:**
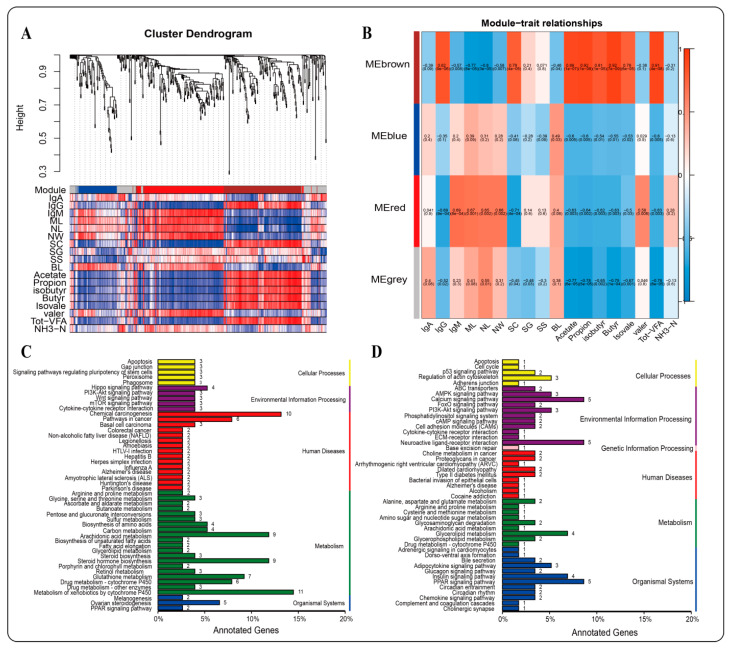
WGCNA analysis. (**A**): Gene-phenotype cluster; (**B**): WGCNA module diagram; (**C**): KEGG enrichment diagram (MEbrown); (**D**): KEGG enrichment diagram (MEred).

**Figure 8:**
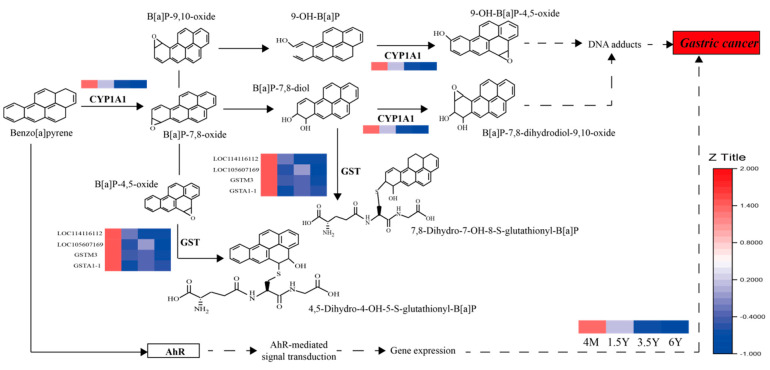
Immune-related pathways. Note: The heat map in the pathway represents gene expression at four ages and the redder the color, the higher the gene expression. CYP1A1: *gene-CYP1A1*; GST: *gene-GSTA1-1, gene-GSTM3, LOC114116112, LOC105607169*; AhR: aryl hydrocarbon receptor. 4M: 4 months; 1.5Y: 1.5 years; 3.5Y: 3.5 years; 6Y: 6 years.

**Figure 9:**
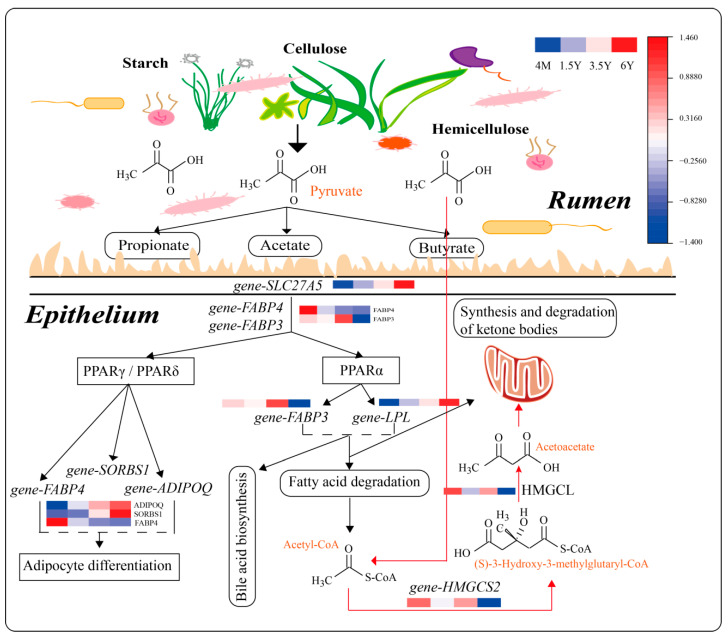
PPAR signaling pathway. Note: The heat map in the pathway represents gene expression at four ages and the redder the color, the higher the gene expression.

Based on the results of RNA transcriptome analysis, weighted gene coexpression network analysis (WGCNA) was further performed on 1006 DEGs, and four gene modules were found to have different degrees of association with traits (immune indicators, VFAs, rumen epithelial structure) of Tibetan sheep (Figure 7A,B). There was a strong correlation between the MEbrown module and VFAs, IgG and SC. Further KEGG functional enrichment analysis of genes in the MEbrown module showed that the genes were mainly enriched in metabolic processes (38.66%), followed by human diseases (18.49%) and organismal systems (16.80%). In metabolic function, genes are mainly enriched in the metabolism of xenobiotics by cytochrome P450 (ko00980). Second, in steroid hormone biosynthesis (ko00140) and arachidonic acid metabolism (ko00590), organismal systems were enriched in the PPAR signaling pathway (ko03320) (Figure 7C). In human diseases, differentially expressed genes were mainly enriched in chemical carcinogenesis (ko05204), in which *gene-CYP1A1*, *gene-GSTA1-1*, *gene-GSTM3* and other genes were significantly enriched and involved in the immune process of the body (Figure 8). The MEred module is associated with rumen epithelial muscle layer and papilla development, and the main annotations are organismal systems (27.27%), environmental information processing (21.21%), human diseases (20.20%) and metabolism (17.17%) (Figure 7D). Further enrichment analysis showed that most of the genes were enriched in the calcium signaling pathway (ko04020), glycerolipid metabolism (ko00561) and PPAR signaling pathway (ko03320). Among them, genes such as *ADIPOQ*, *CPT1B*, *LPL*, *SLC27A5* and *SORBS1* were involved in the regulation (Figure 9).

## 3. Discussion

Tibetan sheep can live in the harsh plateau environment and maintain population reproduction, which is closely related to the interaction between the host genome and the characteristics of tissues and organs [25]. The nonspecific humoral immune indices (IgA, IgG, IgM) of ruminants can reflect the immune health status of plateau animals [26,27]. This study found that with increasing age, the content of highly active IgM in Tibetan sheep increased significantly and played the role of clearing pathogens [28] to improve body immunity. However, IgA mediates the intestinal mucosal immune system [29], and the IgA content of Tibetan sheep aged 1.5 and 3.5 years is higher, indicating better intestinal barrier function at this stage. VFAs are the main energy material of ruminants [30], and the content of VFAs is the highest in lambs aged 4 months, which may be because 4-month-old Tibetan sheep have not been fully weaned and are in the mode of lactation and grazing feeding. In addition to the fermentation of forage, they also ferment the breast milk they eat [31], thus producing a large amount of VFAs, and a high concentration of butyric acid plays a role in enhancing the intestinal barrier function of 4-month-old lambs [31]. Second, the concentration of VFAs is the highest at 1.5 and 3.5 years old, when the rumen of Tibetan sheep is mature, their feeding speed and feed intake reach the peak period, and the rumen fermentation capacity is strong, which ensures efficient production performance and better adaptation to the plateau environment. However, the rumen fermentation ability of 6-year-old sheep was decreased, which resulted in a decrease in VFAs content. In addition, studies have reported that the lower the A/P ratio is, the higher the energy utilization rate of the host [32]. Four-month-old lambs are in the stage of rapid growth and development, and the energy utilization rate should be improved to meet their own growth needs in the plateau environment. VFAs not only serve as energy material but also affect the development of the rumen epithelial structure [33,34]. This study found that with increasing age, the width of the ruminal papilla of Tibetan sheep increased, which expanded the contact area of chyme and ensured that more VFAs were transported and absorbed into the blood for energy supply. Under electron microscope and transmission electron microscope, it was observed that the stratum corneum of 6-year-old Tibetan sheep was seriously shed, the cells in the stratum corneum showed accelerated migration and the intercellular space was gradually enlarged. These results indicate that the physical barrier structure of the rumen epithelium of Tibetan sheep will be damaged with age, the connection between the stratum corneum and stratum granulosum becomes loose, and a large number of keratinized granules exist, thus affecting the absorption, metabolism and transport of VFAs [35]. A large number of desmosomal junctions between cells in the stratum granulosum of the rumen epithelium at 1.5 and 3.5 years of age formed a compact barrier structure, resulting in reduced stratum granulosum thickness and playing an important role in maintaining the integrity of the metabolite concentration gradient throughout the rumen wall [36]. The basal layer is an important energy metabolic layer in the rumen [37]. Transmission electron microscopy showed that there were more mitochondria in the basal layer of the rumen of 1.5 and 3.5-year-old Tibetan sheep, which were involved in regulating the TCA (Tricarboxylic acid cycle) cycle, providing energy for cells [38]. Therefore, we conclude that 1.5 and 3.5-year-old Tibetan sheep can better maintain the energy requirements of epithelial cells. To reveal the molecular mechanism of such metabolic and morphological differences, RNA-seq analysis was further performed on rumen epithelial tissues.

RNA-seq is a method applied to the analysis of transcriptional status and the discovery of functional genes [39,40]. In this study, 1006 DEGs were found in rumen epithelial tissues of Tibetan sheep at different ages, among which five differentially coexpressed genes were found. *NOS2* is involved in the regulation of various biochemical pathways and energy metabolism and acts as a metabolic enzyme in immune and nonimmune cells [41]. *SULT1C2* plays an important role in toxin clearance and lipid metabolism disorder [5,42], and these genes are highly expressed in 3.5-year-old Tibetan sheep, indicating that Tibetan sheep have the highest immune, digestive and metabolic functions in the adult stage. Further functional annotation analysis of these differentially expressed genes showed that they were mainly enriched in cellular processes and involved in regulating the development of rumen epithelial cells, which was consistent with the research results of Lin et al. [5] and could better explain the reasons for the differences in the morphological structure of the rumen epithelium in Tibetan sheep at different ages. Second, it was enriched in metabolic process and biological regulation, which results in the different adaptability of Tibetan sheep to the plateau environment at different ages. Enrichment analysis of biological process (BP) shows that the 4 months_vs._1.5 years and 4 months_vs._6 years groups are mainly enriched in the oxidation-reduction process, in which the enriched gene *GPX1* is involved in the regulation of metabolic homeostasis under oxidative stress [42]. Second, compared with the 4 months_vs._1.5 years group, some upregulated genes were mainly enriched in ISG15-protein conjugation, and negative regulation of the viral genome replication pathway, which are mainly related to the immune regulation of the body. Among them, *ISG15* is involved in the disease occurrence process of the body [43]. The enriched gene *HERC5* is a member of the HERC ubiquitin ligase family, and this protein is a regulator of antiviral immune response [44], indicating that the disease resistance of Tibetan sheep is improved from 4 months to 1.5 years old. The 1.5 Y_vs._3.5 years and 3.5 years_vs._6 years groups were mainly enriched in skeletal muscle thin filament assembly and mesenchyme migration, which are related to the contraction ability of epithelial cells. Among them, *ACTG2* is involved in the regulation of cell movement and maintenance of the cell skeleton, which is related to gastrointestinal peristalsis [45]. It is highly expressed in 3.5-year-old Tibetan sheep, indicating that the creep force of the stomach is improved. In the above morphological structure, it was also found that the muscle fibers of the rumen muscularis at 3.5 years old are more developed, which is related to the contraction and peristalsis of the rumen muscularis.

KEGG function analysis showed that rumen epithelial function was mainly enriched in arachidonic acid metabolism (ko00590) and glutathione metabolism (ko00480) from 4 months to 1.5 years of age, which was related to the resistance to peroxide damage and may have boosted the body’s immunity [46]. Because of the high intensity of ruminal development from 4 months to 1.5 years of age, the immunity of the rumen epithelium may be improved. The ages 1.5 and 3.5 years old are the stages of youth and adulthood, functions are mainly enriched in vascular smooth muscle contraction (ko04270), tight junction (ko04530) and other pathways, and among them, the enriched gene *ACTG2* is the main component of the contractile organ, which improves the contraction and peristalsis of rumen muscle layer [45]. Second, *IGSF5* is involved in the tight junctions of the rumen epithelium [47], which improves the epithelial barrier. In the old age stage from 3.5 to 6 years old, the genes were mainly enriched in arachidonic acid metabolism (ko00590), metabolism of xenobiotics by cytochrome P450 and steroid hormone biosynthesis, which is related to resistance to peroxide damage [46], and immunity was decreased in the old age of 6 years. In addition, it was enriched in the butanoate metabolism pathway, which is a ketogenic metabolism pathway that provides lipid-derived energy for various organs. It has been reported that *ACAT1* and *HMGCS2* isoforms are highly correlated with rumen ketogenesis [48,49]. As shown in Figure 9, the expression of *HMGCS2* is consistent with the concentration of VFAs, and they are involved in the generation and metabolism of ketone bodies through the pyruvate-acetyl-CoA pathway. In addition to 4-month-old lambs, 3.5-year-old sheep are also significantly enriched in ketogenic metabolic pathway for energy supply to better adapt to the plateau environment. In the 4 months_vs._6 years group, the DEGs were mainly enriched in glutathione metabolism (ko00480) and metabolism of xenobiotics by cytochrome P450 (ko00980), and the enriched genes were downregulated, thus leading to a decrease in immunity.

WGCNA showed that the module genes related to rumen development and fermentative metabolism were mainly enriched in metabolism of xenobiotics by cytochrome P450 (ko00980) and chemical carcinogenesis (ko05204), which is involved in the regulation of gastrointestinal diseases (Figure 9). The expression of the enriched gene *CYP1A1/GSTM* decreases with age, and studies have found that these two genes are related to rumen toxin clearance [50], indicating that the probability of gastrointestinal diseases increases in 6-year-old Tibetan sheep, while the immunity of 4-month-old Tibetan sheep is improved through breast milk due to incomplete weaning (lactation + grazing). Second, studies have found that steroid hormones mediate various important developmental and physiological functions of different organs [51], while arachidonic acid is mainly found in glycerolipids [52], and their enrichment may have a certain regulatory effect on the rumen epithelial development and phospholipid membrane of the upper cortex. In addition, we found that module genes related to VFAs and rumen epithelial structure were enriched in the PPAR signaling pathway (Figure 9). Cellulose, hemicellulose and starch in herbage are fermented by rumen microbiota to pyruvate and VFAs, then through *SLC27A5* transshipment, further involved in fatty acid degradation pathway, by acetyl-CoA to participate in the generation and degradation process of ketone bodies, to provide energy for Tibetan sheep rumen epithelial cells and to satisfy energy demands in the plateau environment. In addition, the PPARγ pathway is involved in adipose differentiation. The expression levels of *ADIPOQ* and *SORBS1* increase with age, and *SORBS1* is highly expressed and increases with age. Studies have found that it is involved in the glucose transporter 4 (*GLUT4*)-related pathway [53], which may be involved in the regulation of energy metabolism.

## 4. Materials and Methods

### 4.1. Experimental Design and Sample Collection

Tibetan sheep grazing in Haiyan County, Haibei Prefecture, Qinghai Province were selected as the research objects, at an altitude of 3500 m. Twenty Tibetan sheep (Euler type, ♀) were randomly selected from the flock of the same herdsman. They were 4 months old (n = 5, lamb), 1.5 years old (n = 5, young), 3.5 years old (n = 5, adult) and 6 years old (n = 5, old), and they were all in local, traditional, natural grazing management modes, in which 4-month-old lambs follow their mothers to graze. Samples were collected in August 2020, and forage species and nutrient levels in the pasture are shown in Table 3.

The jugular blood of Tibetan sheep was collected in a vacuum tube and centrifuged (5000× *g*, 4 °C) for 20 min, and the serum was separated and stored at −20 °C for the determination of immune indices. According to the ethical approval requirements of the ethics committee and the local traditional slaughter and sampling methods, the rumen organs were removed within 10 min after slaughter, and a large piece of rumen epithelial tissue (abdominal sac) was cut and washed with normal saline. One part of the sample was clipped and placed into a cryopreservation tube for rapid freezing for transcriptome sequencing, and the other part was fixed in 4% paraformaldehyde and 2.5% glutaraldehyde for hematoxylin-eosin staining (HE) section and transmission electron microscope (TEM) section. A total of 50 mL of rumen contents was collected and stored in an ice box at −20 °C for determination of the fermentation metabolic parameters, VFAs and NH_3_-N.

### 4.2. Serum Immune Index Determination

The serum concentrations of immunoglobulin A (IgA), immunoglobulin M (IgM) and immunoglobulin G (IgG) were determined using the enzyme-linked immunosorbent assay method (ELISA, Nanjing Jiancheng Institute of Bioengineering Ltd., Nanjing, China), and the assay was performed with a Microplate Reader (Thermo Fisher Scientific, Waltham, MA, USA).

### 4.3. Determination of the Rumen Fermentation Parameters

Rumen fluid was centrifuged at 5400 rpm for 10 min, and the supernatant was extracted according to the method of Liu et al. [4], and the composition and content of VFAs were determined by Shimadzu gas chromatograph (GC-2010 plus). Rumen fluid was centrifuged at 3500~4000 r/min for 10 min, and NH_3_-N content was determined according to Chaney et al. [54].

### 4.4. Micromorphological Structure of the Rumen

The rumen abdominal sac tissue was cut into 1 cm × 1 cm tissue blocks and fixed in 4% paraformaldehyde. After fixation for 24 h, the sections were dehydrated, made transparent, waxed, embedded, sectioned and stained with hematoxylin and eosin to make HE sections; hematoxylin stains the nucleus blue-purple, and eosin stains the cytoplasm pink. Rumen muscularis thickness, papillary height, papillary width, stratum corneum thickness, stratum granulosum thickness, stratum spinosum thickness and basal layer thickness were measured using the CaseViewer slice analysis system.

The rumen epithelium was cut into tissue blocks less than 1 mm^3^, fixed in 2.5% glutaraldehyde solution for 24 h, rinsed with PBS, and postfixed in 1% osmic acid for 2 h. After rinsing with double steaming water, the tissue was dehydrated with serial alcohol and embedded in 100% resin. Ultrathin microsections (Leica, EM-UC6, Wetzlar, Germany) (60–100 nm) were counterstained with 3% uranyl acetate and lead citrate, and the ultrastructure was observed under a transmission electron microscope (Hitachi, H-7500, Tokyo, Japan).

### 4.5. RNA Extraction and Transcriptome Sequencing

The Trizol reagent method (DP762-T1C) was used to extract total RNA from rumen epithelial tissues of Tibetan sheep, and a Nanodrop 2000 was used for concentration detection. Integrity was checked using Agient2100, LabChip GX (Model Platinum Elmer LabChip GX). The cDNA Library was constructed using the VAHTS Universal V6 RNA-seq Library Prep Kit for Illumina^®^ kit (NR604-02), and the procedure of the kit was strictly followed. The product was further purified using a VAHTSTM DNA Clean Beads Kit (N411-03). The constructed libraries were sequenced using an Illumina NovaSeq6000 (San Diego). Bioinformatics analysis was conducted on BMKCloud, and clean data were obtained after data filtering of raw data off the machine. Mapped data were obtained by sequence alignment of clean data with the specified reference genome Ovis_aries (Oar_rambouillet_v1.0.Ovis_aries) through HISAT [55]. The reads on the pair were assembled using StringTie [56], and FPKM [57] (fragments per kilobase of transcript per million fragments mapped) was used as an index to measure the expression level of transcripts or genes. The DESeq2 [58] data analysis method was used to analyze differentially expressed genes. Fold change (FC) represents the ratio of expression between two samples (groups); false discovery rate (FDR) was obtained by correcting the *p*-value of difference significance. Having a fold change ≥ 2 and FDR < 0.01 were used as the screening criteria for differentially expressed genes. Gene Ontology (GO) and Kyoto Encyclopedia of Genes and Genomes (KEGG) functional enrichment analyses were conducted using GOseq [59].

### 4.6. Data Analysis

Excel 2016 was used to sort out the data, and IBM SPSS Statistics (V.22.0) was used to analyze the significance of serum immune indexes, rumen morphological data and fermentation parameters. Single-factor ANOVA analysis and Duncan’s multiple test were performed, *p* < 0.05 was considered a significant difference and the results were expressed as the Mean value and Standard Error of Mean (SEM). Weighted gene co-expression network analysis (WGCNA) [60] was performed to understand the link between the host transcriptome (mRNA) and the Tibetan sheep phenotypic traits (fermentation parameters and morphological indexes).

## 5. Conclusions

This study revealed the differences in rumen development and metabolism of Tibetan sheep at different ages. With increasing age, the width of the rumen papilla increased significantly, expanding the contact area of chyme. Among them, the rumen epithelial layer of young sheep (1.5 years old) and adult sheep (3.5 years old) is closely connected, the basal layer has a large number of mitochondria, which are involved in energy supply, and the rumen fermentation metabolism function is complete. RNA-seq revealed that epithelial differential gene enrichment in lambs (4 months old) and older sheep (6 years old) was involved in regulating metabolic immunity. Epithelial differential genes are enriched in cell contraction and immune barrier function and play a role in energy metabolism in the ketogenic metabolic pathway in young and adult sheep. Comprehensive analysis revealed that genes related to epithelial morphology and VFAs metabolism were enriched in the metabolism of xenobiotics by cytochrome P450 and PPAR pathways, which regulate rumen immunity and fermentative metabolism in different stages of Tibetan sheep, providing a new approach for the study of plateau adaptability.

## Figures and Tables

**Table 1:** Rumen fermentation parameters of Tibetan sheep at different ages.

	4 Months	1.5 Years	3.5 Years	6 Years	*p_*Value**
Acetic acid (mmol/L)	119.66 ± 1.68 ^a^	30.72 ± 5.54 ^c^	69.08 ± 4.52 ^b^	19.30 ± 3.15 ^d^	<0.00
Propionic acid (mmol/L)	24.63 ± 0.68 ^a^	3.26 ± 0.87 ^c^	10.90 ± 1.85 ^b^	2.70 ± 0.25 ^c^	<0.00
Isobutyric acid (mmol/L)	1.90 ± 0.22 ^a^	1.14 ± 0.13 ^c^	1.53 ± 0.20 ^b^	0.79 ± 0.07 ^d^	<0.00
Butyric acid (mmol/L)	18.82 ± 0.52 ^a^	2.00 ± 0.39 ^c^	5.91 ± 0.93 ^b^	1.49 ± 0.32 ^c^	<0.00
Isovaleric acid (mmol/L)	2.24 ± 0.42 ^a^	1.27 ± 0.27 ^c^	1.81 ± 0.26 ^b^	1.27 ± 0.25 ^c^	<0.00
Pentanoic acid (mmol/L)	0.09 ± 0.02 ^bc^	0.05 ± 0.03 ^c^	0.16 ± 0.09 ^b^	0.35 ± 0.04 ^a^	<0.00
Total VFAs (mmol/L)	167.33 ± 1.39 ^a^	38.43 ± 5.36 ^c^	89.39 ± 7.18 ^b^	25.89 ± 3.43 ^d^	<0.00
A/P	4.86 ± 0.18 ^b^	10.33 ± 4.86 ^a^	6.45 ± 0.90 ^b^	7.14 ± 0.83 ^ab^	0.023
NH_3_-N (mg/100 mL)	6.26 ± 0.04 ^d^	7.23 ± 0.05 ^c^	12.26 ± 0.10 ^a^	7.45 ± 0.04 ^b^	<0.00

Note: Different superscript lowercase letters indicate significant differences on the same line, *p* < 0.05; A/P: acetic acid/propanoic acid.

**Table 2:** Structure of rumen epithelium at different ages (μm).

	4 Months	1.5 Years	3.5 Years	6 Years	*p*-Value
ML	1644.00 ± 73.60 ^d^	1925.24 ± 56.55 ^c^	2067.68 ± 133.51 ^b^	2293.48 ± 126.21 ^a^	<0.00
Lp	1219.98 ± 180.70 ^b^	1846.56 ± 44.77 ^a^	2020.96 ± 215.65 ^a^	1962.40 ± 111.19 ^a^	<0.00
Wp	368.68 ± 13.85 ^c^	400.46 ± 22.27 ^b^	403.36 ± 13.73 ^b^	536.98 ± 25.06 ^a^	<0.00
SC	58.72 ± 3.07 ^c^	44.16 ± 1.41 ^b^	43.42 ± 2.07 ^b^	21.52 ± 2.71 ^a^	<0.00
SG	27.22 ± 3.83 ^ab^	14.86 ± 0.79 ^c^	23.44 ± 1.27 ^b^	29.68 ± 4.80 ^a^	<0.00
SS	54.82 ± 4.39	50.32 ± 4.74	50.26 ± 3.60	52.72 ± 3.32	0.265
BL	19.44 ± 1.22 ^b^	21.34 ± 2.48 ^b^	18.72 ± 1.65 ^b^	24.62 ± 2.80 ^a^	0.002

Note: Length of the papilla (Lp); width of the papilla (Wp); stratum corneum (SC); stratum granulosum (SG); stratum spinosum (SS); basal layer (BL); muscular layer (ML). Different superscript lowercase letters indicate significant differences on the same line, *p* < 0.05.

**Table 3:** Forage species and nutrient levels.

Nutrients (DM Basis)		Dominant Forage Species
CP (%)	10.06	*Poa pratensis L*
EE (%)	3.77	
Ash (%)	4.55	*Elymus nutans Griseb*
NDF (%)	70.11	
ADF (%)	36.17	*Agropyron cristatum (L.) Gaertn*
HCEL (%)	33.94	
Ca (%)	11.50	*Stipa aliena Keng*
P (%)	0.65	
Aboveground biomass (g/m^2^)	343.52	*Potentilla bifurca Linn.*
Grass height (cm)	16.12	

Note: Crude protein (CP); crude fat (EE); dry matter (DM); crude ash (Ash); neutral detergent fiber (NDF); acid detergent fiber (ADF); hemicellulose (HCEL).

## Data Availability

The datasets presented in this study can be found in online repositories. The names of the repository/repositories and accession numbers can be found below: Sequence Read Archive (SRA): SRR21874478-SRR21874492, SRR18466104-SRR18466108.

## Supplementary Materials

The following supporting information can be downloaded at: https://www.mdpi.com/article/10.3390/ijms232416078/s1, Figure S1. GO classification.
