# Supplementary figures and images for: Rumen Epithelial Development- and Metabolism-Related Genes Regulate Their Micromorphology and VFAs Mediating Plateau Adaptability at Different Ages in Tibetan Sheep
Source: Int J Mol Sci. 2022 Dec 16;23(24):16078. doi: 10.3390/ijms232416078 (PMC9786296; doi:10.3390/ijms232416078)

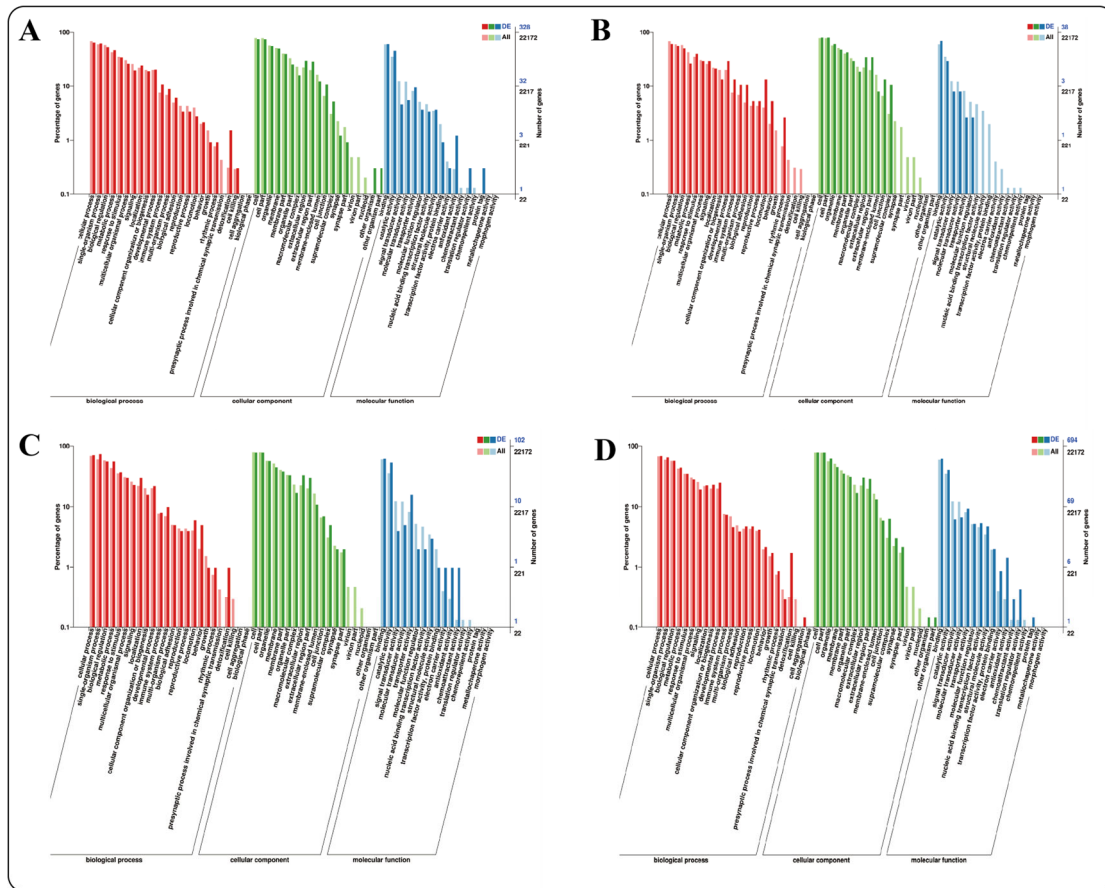

**Figure S1.** GO classification. **A:** 4M\_vs\_1.5Y; **B:** 1.5Y\_vs\_3.5Y; **C:** 3.5Y\_vs\_6Y; **D:** 4M\_vs\_6Y

Supplement: Supplementary file 1 [file ijms-23-16078-s001.zip › ijms-2056190-supplementary.pdf]
